# Callous-Unemotional Traits Moderate Anticipated Guilt and Wrongness Judgments to Everyday Moral Transgressions in Adolescents

**DOI:** 10.3389/fpsyt.2021.625328

**Published:** 2021-03-05

**Authors:** Margarida Vasconcelos, Essi Viding, Catherine L. Sebastian, Susana Faria, Pedro R. Almeida, Óscar F. Gonçalves, Rui A. Gonçalves, Adriana Sampaio, Ana Seara-Cardoso

**Affiliations:** ^1^Psychological Neuroscience Laboratory, Psychology Research Centre, School of Psychology, University of Minho, Braga, Portugal; ^2^Division of Psychology and Language Sciences, University College London, London, United Kingdom; ^3^Department of Psychology, Royal Holloway University of London, London, United Kingdom; ^4^Department of Mathematics, University of Minho, Braga, Portugal; ^5^Faculty of Law, Interdisciplinary Research Centre on Crime Justice and Security, School of Criminology, University of Porto, Porto, Portugal; ^6^Proaction Laboratory, Faculty of Psychology and Educational Sciences, University of Coimbra, Coimbra, Portugal; ^7^School of Psychology, Campus de Gualtar, University of Minho, Braga, Portugal

**Keywords:** Callous-unemotional (CU) traits, psychopathy, adolescence, moral emotion, moral judgement

## Abstract

Callous-unemotional (CU) traits observed during childhood and adolescence are thought to be precursors of psychopathic traits in adulthood. Adults with high levels of psychopathic traits typically present antisocial behavior. Such behavior can be indicative of atypical moral processing. Evidence suggests that moral dysfunction in these individuals may stem from a disruption of affective components of moral processing rather than from an inability to compute moral judgments *per se*. No study to date has tested if the dissociation between affective and cognitive dimensions of moral processing linked to psychopathic traits in adulthood is also linked to CU traits during development. Here, 47 typically developing adolescents with varying levels of CU traits completed a novel, animated cartoon task depicting everyday moral transgressions and indicated how they would feel in such situations and how morally wrong the situations were. Adolescents with higher CU traits reported reduced anticipated guilt and wrongness appraisals of the transgressions. However, our key finding was a significant interaction between CU traits and anticipated guilt in predicting wrongness judgments. The strength of the association between anticipated guilt and wrongness judgement was significantly weaker for those with higher levels of CU traits. This evidence extends our knowledge on the cognitive-affective processing deficits that may underlie moral dysfunction in youth who are at heightened risk for antisocial behavior and psychopathy in adulthood. Future longitudinal research is required to elucidate whether there is an increased dissociation between different components of moral processing from adolescence to adulthood for those with high psychopathic traits.

## Introduction

The term callous-unemotional (CU) refers to a constellation of personality traits that include blunted affect, lack of empathy and remorse, uncaring behavior and disregard for others' feelings and well-being (1–4). CU traits have received increased attention in the last decades. Its presence seems to distinguish a cohort of youth who exhibit instrumental and planned violence, and who display a subtype of conduct disorder that is more severe, more stable and more resistant to treatment [e.g., (5–7)]. High levels of CU traits are thought to contribute to the development of a more persistent and aggressive type of antisocial behavior in youth, found across forensic [e.g., (8, 9), community [e.g., (1, 5)], and mental health samples [e.g., (10)]. Even in the absence of conduct disorder, high levels of CU traits have been associated with higher risk for disruptive behavior [e.g., (5, 11, 12)]. Indeed, longitudinal data indicate that CU traits add to the prediction of serious and persistent criminal behavior in boys, over and above the presence of conduct disorder symptoms and oppositional defiant problems (13).

Contemporary perspectives on human morality have emphasized the role of emotions on moral reasoning and behavior (14, 15). Moral emotions—i.e., “emotions that respond to moral violations or that motivate moral behavior” [(16), p. 853]—in particular seem to have a prominent role in moral judgment—i.e., our ability to tell right from wrong (15, 17–19). It has been hypothesized that the experience of moral emotions, such as guilt, may help people to identify the moral implications of their judgments and behaviors and that the anticipation of moral emotions may support regulation of appropriate social behavior [see (20) for a recent review]. Studies suggest that the lack of adherence to moral and social norms may be rooted in emotional processes rather than moral reasoning processes (21, 22). For example, guilt proneness, i.e., the predisposition to experience negative feelings about personal wrongdoings, consistently predicts appropriate moral behavior (23). Also, guilt aversion—i.e., guilt evoked when an agent believes he had failed/hurt others based on failing their expectations (24)—is suggested to strongly motivate people's choices during cooperative efforts such as games, plausibly playing a role in moral judgments (25–27). Feelings of guilt are thought to provide immediate and salient feedback on either executed or imagined behavior (21). Therefore, the anticipation of guilt about committing a wrongdoing can work as a “brake” that curbs antisocial behavior.

CU traits are thought to be the precursor of affective and interpersonal psychopathic traits in adulthood (28). Extant evidence suggests that moral dysfunction in adults with high levels of psychopathic traits may stem from a disruption of the affective and motivational components of moral processing rather than from an inability to compute moral judgments *per se* (29). For example, high levels of psychopathic traits seem to be associated with reduced propensity to feel moral emotions (i.e., guilt) and reduced difficulty in judging actions in moral dilemmas (30, 31), but not with endorsement of such actions [(30, 32) but see (33) for an exception]. Concomitantly, psychopathic traits are associated with reduced responses in affective brain regions during moral processing despite apparent intact moral judgment ability (34, 35). In parallel, a growing body of research has been showing a dissociation between affective and cognitive empathy impairments in individuals with high level of psychopathy (e.g., 30, 36–38). Individuals with high levels of psychopathy seem to have intact cognitive empathy, i.e., are able to infer and describe what others feel, but show impairments in affective empathy, i.e., fail to resonate with others' feelings (30, 36–38). The same pattern of dissociation between affective and cognitive empathic processes has been identified in children with high levels of CU traits (39–41).

Whilst accumulating evidence suggests a dissociation between affective and cognitive components of moral processing in adults with high levels of psychopathy, it is not clear whether this dissociation is already at play during development. A few studies have now inspected correlates of moral dysfunction associated with CU traits. For example, akin to adults with high psychopathic traits (18, 42), children and adolescents with high levels of CU traits present difficulties in distinguishing between moral and conventional transgressions (43, 44). They report that, if given permission from a figure of authority, it is as acceptable to break a conventional societal rule, such as leaving the classroom, as it is to break a moral rule such as not to hit someone. This failure to distinguish between the two types of transgressions is thought to reflect diminished emotional resonance to the harm caused to the victim. More recently, Fragkaki et al. (45) found that high levels of CU traits were associated with lower feelings of guilt when individuals were imagining themselves committing antisocial acts. Neuroimaging research suggests that the atypical moral behavior that children and youth with high CU traits display may stem from dysfunction in brain regions implicated in affective processing, namely the amygdala and the ventromedial prefrontal cortex [vmPFC; for a review see, (46)]. For example, adolescents with higher CU traits show a negative association between amygdala responses and ratings of moral violations severity (47) and reduced amygdala response during implicit moral judgement (48). Additionally, they show a reduced connectivity between amygdala and vmPFC during both explicit and implicit moral judgment (47, 48). Overall, the evidence suggests that youths with high CU and adults with high psychopathic traits seem to be remarkably similar in terms of their behavioral and neurobiological impairments associated with moral judgments [for a review see (49)].

No study to date has tested if the dissociation between affective (i.e., anticipated guilt) and cognitive (i.e., wrongness judgments) dimensions of moral behavior observed in adults with high levels of psychopathy is also linked to CU traits during development. This is important, as it will help us gain a more precise picture of the cognitive-affective processing deficits that may underlie atypical moral processing in youth who are at heightened risk for antisocial behavior and psychopathy in adulthood. Moreover, extant research has been mostly conducted in populations with disruptive behavior disorders. However, an increasing number of studies have advocated the importance of studying CU traits in community samples because these traits are almost always accompanied with some conduct disturbance, predict poorer outcomes and also because many children with clinically significant needs do not receive treatment (50–53). In youth, CU traits are linked to a constellation of problems indicative of a subclinical variation of antisocial behavior, such as increased risk-taking behavior, hyperactivity, and poor peer relationships (13, 53–55). Akin to psychopathic traits in adulthood (56), evidence suggests CU traits to be continuously distributed in the general population (57). Research with non-forensic adult samples has revealed similar associations (comparatively with forensic samples) between psychopathic traits and abnormal moral behavior (30, 31, 58).

In the present study, we developed a novel animated cartoon task depicting first-person everyday harm-based moral transgressions and asked adolescents from the community to report: (1) how they would feel in such situations, and (2) how wrong such actions would be. Importantly, in this task, and contrary to most of the tasks used in morality research, we used everyday scenarios to assess moral decision making rather than life and death situations which are unlikely to be encountered in everyday life. All scenarios portrayed situations that involve harming others for personal gain. Scenarios were carefully designed to guarantee that they corresponded to moral transgressions but also that their content was developmentally appropriate. We predicted that CU traits would be negatively associated with variance in anticipated guilt to everyday moral transgressions but not with variance in moral wrongness judgments (both in terms of participants' ratings and response times [RTs]). Importantly, we predicted that CU traits would be linked with a higher dissociation between anticipated guilt and moral wrongness appraisals.

## Materials and Methods

### Participants

Forty-seven typically-developing male adolescents with ages ranging from 15 to 18 years (*M*_age_ = 16.19 years; *SD* = 0.89) took part in this study. We focused on male adolescents for a number of reasons. Male adolescents present higher levels of CU traits, antisocial behavior, delinquency and commit more crimes than girls (59). Plus, the majority of research on moral processing in adults with high levels of psychopathy has focused on adult males. A male adolescent sample would allow us a direct comparison of results with the literature on adult populations whilst avoiding the need to include an extra gender variable which would require a much larger sample. Participants had no history of substance abuse, neurological/psychiatric disorders, or other clinically relevant diagnoses. Participants filled in questionnaires and performed a moral transgressions experimental task on a laptop computer in a single, individual session at their school. Participants and their parents provided written informed consent before taking part in the study. The study was approved and conducted in full accordance with the guidelines set by the Ethics Committee for Health and Life Sciences of University of Minho.

### Materials

#### Inventory of Callous-Unemotional Traits—Self-Report [ICU; (1, 60)]

CU traits were measured using participants' ratings on the Portuguese version of the ICU scale [1; see the validation for the Portuguese population in (60)]. The self-report version of the ICU is a 24-item questionnaire designed to assess callous, unemotional and uncaring traits in children and youth; (Callousness: “I do not care who I hurt to get what I want”; Uncaring: “I feel bad or guilty when I do something wrong”; Unemotional: “I do not show my emotions to others”). The items are rated on a four-point scale ranging from 0 (*not true at all*) to 3 (*definitely true*). Each sub-factor score is computed by summing up its items (some items are reverse-scored) and the total score is obtained by summing up all sub-factors' scores.

#### Everyday Moral Transgressions Task

**Figure 1:**
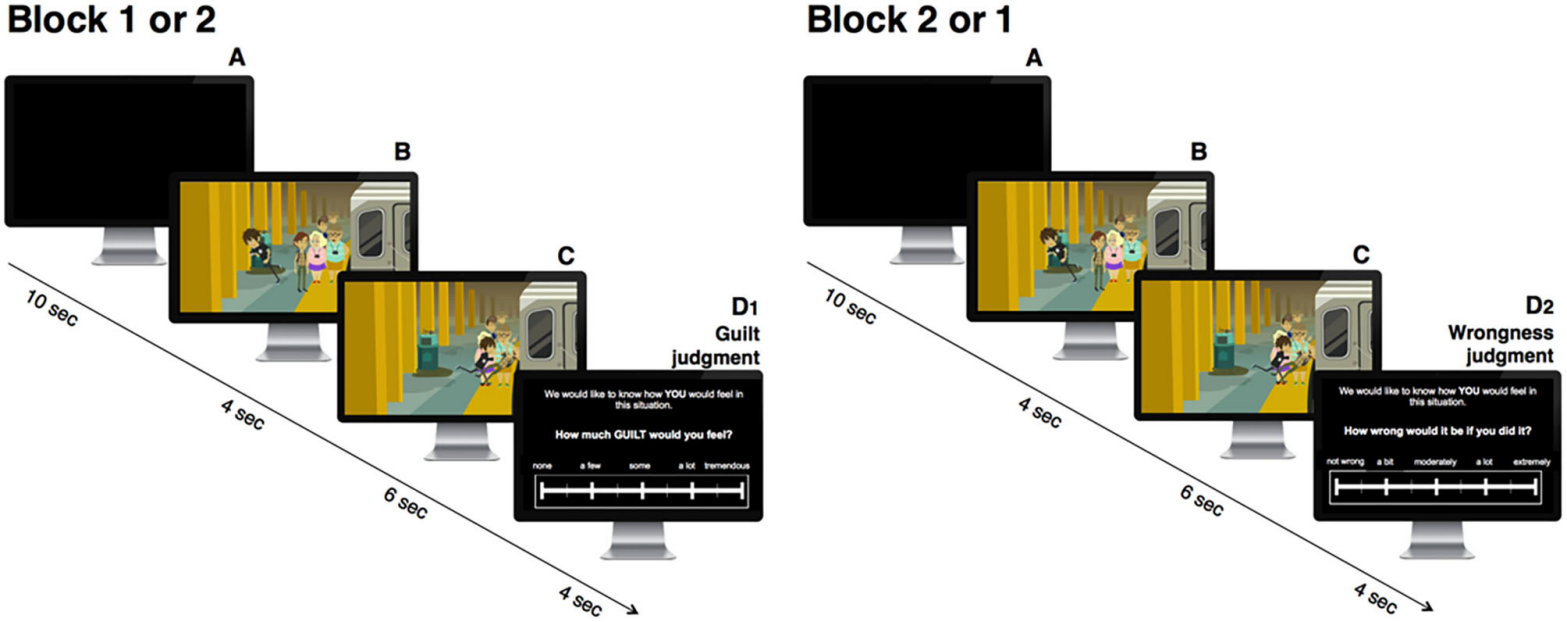
Everyday Moral Transgressions task. Each trial was composed by A to D phases. (A) A black screen was displayed before the beginning of the cartoon. (B) During the first 4 s of the cartoon, the avatar's goal is established (e.g., running to get to the train on time). (C) In the following 6 s, to achieve his goal, the avatar inflicts harm to another person (e.g., pushes someone out of the way, throwing her to the ground). The cartoon is then followed by a question asking participants to anticipate guilt (D1) or perform a wrongness appraisal (D2), depending on the experimental block.

We developed a novel, animated cartoons task depicting everyday moral transgressions, based on Seara-Cardoso et al. (35). In these cartoons, an avatar (a male youth) harms another person in order to achieve a personal goal. Moral transgressions were made as unambiguous as possible by clearly indicating the intentions of the avatar and the consequences of his actions (61). Twenty-seven cartoons depicting everyday moral transgressions were created according to the following structure: (1) the avatar's personal goal/desire is established (4 s); and (2) the avatar harms another person to get his goal/desire (6 s; harm-to-other scenarios, HTO). To ensure that all scenarios were deemed moral transgressions and did elicit feelings of guilt, 27 alternative ending control scenarios were created where, instead of causing harm to another person, the avatar causes harm to himself to achieve the same goal/desire (harm-to-self scenarios, HTS). The task was divided in two blocks, counterbalanced across participants. In one block, participants rated all cartoons on anticipated feelings of guilt (i.e., “How much guilt would you feel?”) on a sliding scale from “*None”* (0) to “*A lot”* (20) passing through a middle stance (“*Some*”). In the other block, participants rated all cartoons on moral wrongness (i.e. “How wrong would this be?”) on a sliding scale from “*Not wrong*” (0) to “*Extremely wrong”* (20) going through a middle stance (“*Somewhat*”) (Figure 1). After watching each cartoon, participants had up to 4 s to complete the rating. Cartoons within each block were randomized. Cronbach's α for guilt and wrongness judgments of moral transgression scenarios were 0.924 and 0.941, respectively. Cartoon video clips were created using GoAnimate (https://www.vyond.com/). The task was programmed in E-Prime (E-Prime 2.0 Build 2.0.10.242). Participants were seated in a comfortable chair at a distance of ~50−100 cm from a computer monitor of 17” and 1,280 × 1,024 resolution (Scenicview A17-3, Fujitsu Siemens) in a quiet, well-illuminated room and used headphones to reduce auditory distraction. Videos were displayed in the upper two thirds of the monitor, and the question and corresponding sliding scale were displayed below. Participants used the computer mouse to provide their answers.

### Statistical Analysis

Then, to check whether the moral transgression cartoons did portray a moral transgression and did elicit feelings of guilt, paired-*t*-tests were conducted to detect significant differences in wrongness and guilt judgments between all pairs of HTO and HTS scenarios. Missing values were excluded. All HTO scenarios, except one (scenario 16, see Supplementary Table 1), presented significantly higher ratings of anticipated guilt and moral wrongness than their alternative HTS scenario (all *t* > 2.50; *p* < 0.01*)*. This scenario was excluded from further analyses to ensure that all the stimuli included portrayed guilt-eliciting moral transgressions.

To examine the relationship between appraisals (anticipated guilt and moral wrongness) of moral transgressions and CU traits (i.e., ICU total score) and between response times during appraisals and CU traits, linear mixed*-effects* models (LMMs) were estimated. LMMs are particularly appropriate for the analysis of nested structured data as is the case of this study. As the data were nested both within participants and within scenarios, participants and scenarios were treated as random effects. Additional LMMs were estimated to test whether CU traits moderated the relationship between feelings of guilt and wrongness ratings, i.e., to test whether levels of CU traits changed the strength of the association between these two variables; and whether CU traits moderated the relationship between RTs in ratings of anticipated guilt and wrongness judgments, which might be considered as a proxy of difficulty in making such judgments. The presence of univariate outliers was checked using the protocol described by Tabachnick and Fidell's (62). The highest RT in anticipated guilt (RT = 3891 ms) was identified as a potential outlier. Removal of this observation had a very small impact on the parameters estimates. We also ran additional models without the observation with the lowest RT in anticipated guilt (RT = 39 ms), given its extremely low value. Removal of both observations had a very small impact on the parameters estimates (see Supplementary Materials for these results). For completeness, we also computed additional models with age as a covariate. Our results indicate that age is positively associated with wrongness ratings and RTs but its inclusion does change the pattern of our results. It should be noted, though, that the limited age range in our sample precludes a definite interpretation of these findings (see Supplementary Materials for these results). Statistical analyses were carried out in R statistical software (63), using nlme package to perform linear mixed effects modeling, and ggplot2 package to construct the graphs. *P-*values lower than 0.05 were considered statistically significant. The raw data supporting the conclusions of this article will be made available by the authors, without undue reservation.

## Results

The means, standard deviations and ranges for all variables are presented in Table 1. HTS scenarios were used only to confirm that all of HTO scenarios portrayed guilt-eliciting moral transgressions. All following analyses relate to moral transgressions (i.e., HTO) scenarios only.

**Table 1:** Characterization of the sample in terms of scores on the ICU, and ratings and response times to the everyday moral transgressions task.

	**Adolescents (*****N*** **=** **47)**					
	***M***		***SD***		**Range**	
**ICU score**	**19.04**		**6.36**		**7 - 36**	
	**HTO scenarios**			**HTS Scenarios**		
	***M***	***SD***	**Range**	***M***	***SD***	**Range**
Wrongness ratings	16.3	4.0	0-20	7.6	6.3	0–20
Guilt ratings	15.7	4.3	0-20	8.8	6.4	0–20
Wrongness RTs	1309.5	736.7	176-3989	1599.1	843.0	39–3955
Guilt RTs	1424.6	722.8	34-3891	1655.9	802.9	257–3993

*ICU, Inventory of Callous-Unemotional traits; HTO, “Harm-To-Other” scenarios; HTS, “Harm-To-Self' scenarios; RTs, Response Times*.

### Are CU Traits Associated With Anticipated Feelings of Guilt and Wrongness Judgments to Everyday Moral Transgressions?

**Figure 2:**
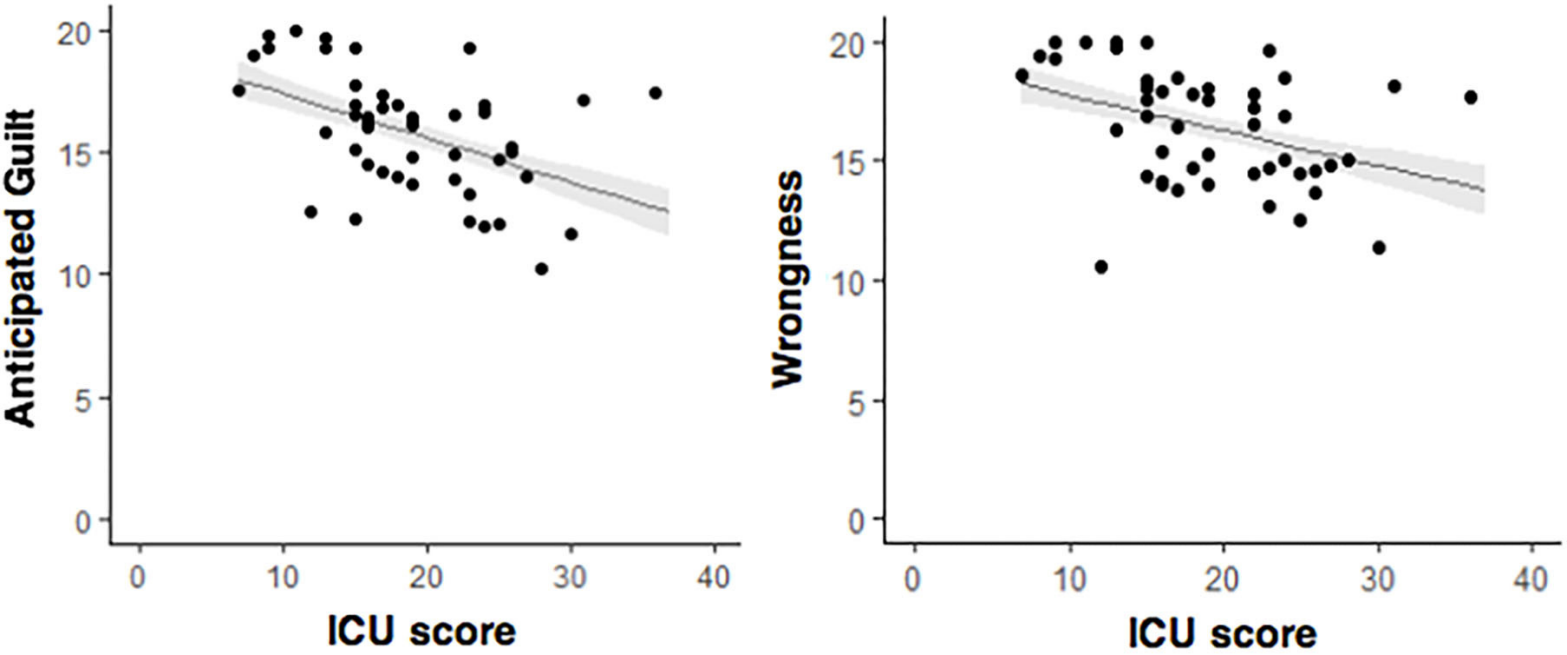
Scatter plots depicting the associations between CU traits (X axes) and anticipated guilt (graph on the left) and wrongness (graph on the right) ratings to moral transgressions. The black lines at each graph illustrate linear effects of CU traits on guilt and wrongness ratings, respectively. The shaded area (in gray) represents the 95% confidence interval of the prediction.

We estimated LMMs with random intercepts for testing the association between anticipated guilt and ICU score, and between wrongness judgments and ICU score. Predictor variables were grand mean centered. Parameter estimates from each model are presented in Table 2 and the relation between variables is illustrated in Figure 2. Higher CU traits were associated with less anticipated guilt (β = −0.18, *p* < 0.001) and less wrongfulness judgments (β = −0.15, *p* < 0.001). The variance in anticipated guilt explained by ICU score was 7.7% and in wrongness judgments was 5.4%, as indicated by the marginal *R*^2^ statistic.

**Table 2:** **Linear mixed models of Anticipated Guilt and Moral Wrongness ratings including the effects of CU traits.**.

	**Guilt ratings**			**Wrongness ratings**		
**Fixed effects**	**β**	**SE**	***p-value***	**β**	**SE**	***p-value***
Intercept	15.81	0.30	< 0.001	16.44	0.29	< .001
ICU score	−0.18	0.02	< 0.001	−0.15	0.02	< .001
**Random effects**	***SD*** **(CI)**			***SD*** **(CI)**		
Scenario (intercept)	1.4 (1.03–1.96)			1.4 (1.03–1.94)		
Participant (intercept)	3.6 (2.52–5.07)			3.3 (2.45–4.52)		
Residual	1.4 (0.13–14.33)			1.3 (0.17–9.78)		
Marginal R^2^	0.077			0.054		
AIC	6686.75			6517.14		

β, unstandardized regression coefficient; SE, standard error; ICU, Inventory of Callous–Unemotional traits; CI, confidence interval; AIC, Akaike Information Criterion.

Two additional LMMs were estimated to test the associations between ICU score and response times in anticipated guilt and wrongness appraisals. Predictor variables were grand mean centered. Parameter estimates from each model are presented in Table 3. ICU scores were positively associated with response times in performing guilt and wrongness appraisals (Guilt: β = 15.38, *p* < 0.001; Wrongness: β = 17.43, *p* < 0.001). That is, those scoring higher on ICU took longer to provide ratings of guilt and wrongness (see Table 3).

**Table 3:** Linear mixed models of Anticipated Guilt and Wrongness RTs including the effects of CU traits.

	**Guilt RTs**			**Wrongness RTs**		
**Fixed effects**	**β**	**SE**	***p-value***	**β**	**SE**	***p-value***
Intercept	1441.96	36.84	< 0.001	1310.18	37.45	< 0.001
ICU Total score	15.38	3.27	< 0.001	17.43	3.26	< 0.001
**Random effects**	***SD*** **(CI)**			***SD*** **(CI)**		
Scenario (intercept)	155.8 (104.86–231.39)			159.8 (107.42–237.58)		
Participant (intercept)	665.0 (34.87–12682.27)			664.4 (15.31–28838.92)		
Residual	251.8 (0.01–441062.82)			251.7 (0.01–649355.72)		
Marginal R^2^	0.012			0.022		
AIC	19176.71			19173.87		

*β, unstandardized regression coefficient; SE, standard error; ICU, Inventory of Callous-Unemotional traits; CI, confidence interval; AIC, Akaike Information Criterion*.

### Do Levels of CU Traits Moderate the Association Between Anticipated Guilt and Wrongness Judgments for Everyday Moral Transgressions?

**Figure 3:**
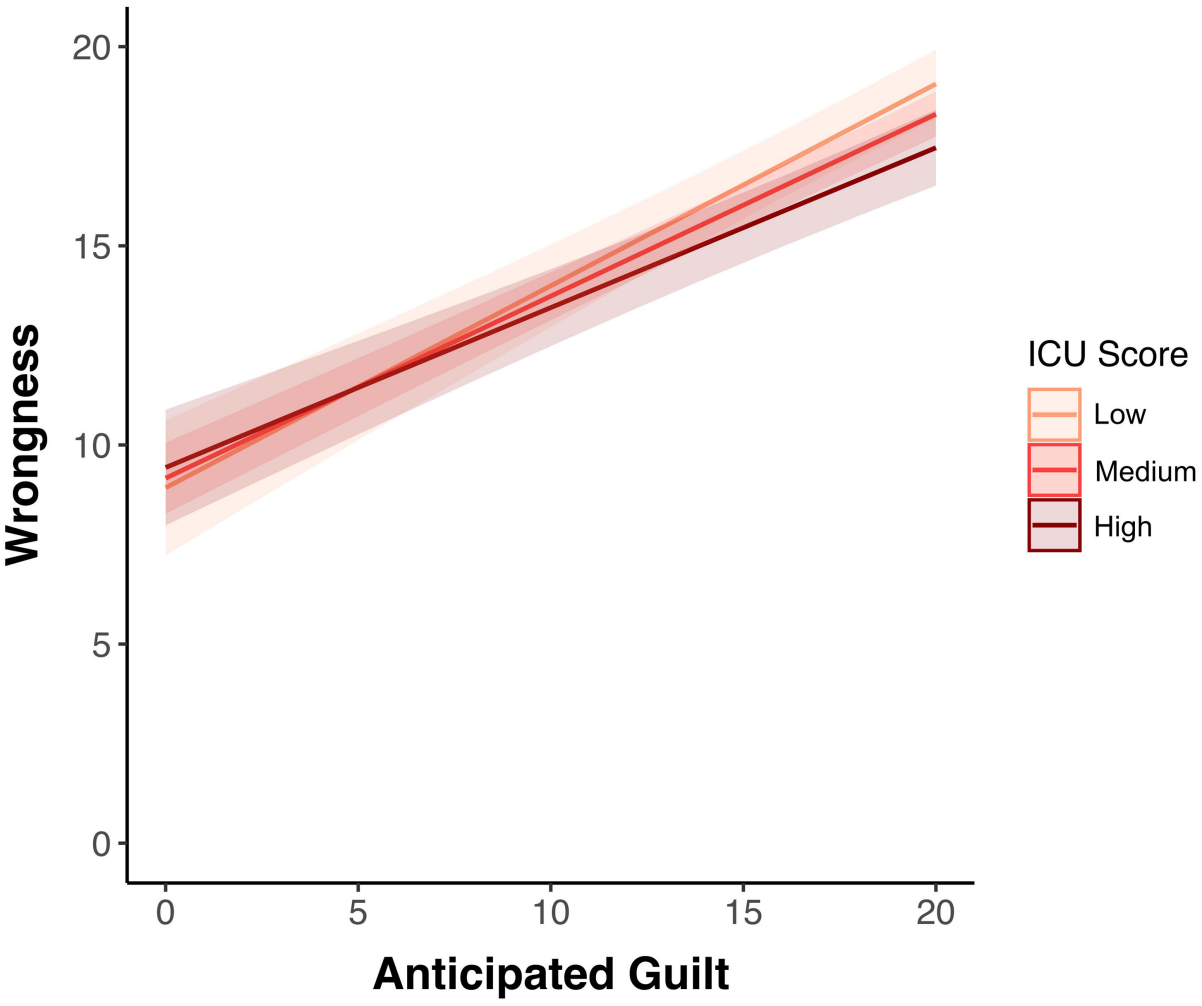
Graph depicts the association between anticipated guilt and wrongness judgments to everyday moral transgressions as moderated by CU traits. The shaded area for each category (i.e., “Low” in light pink, “Medium” in light red, and “High” in dark red) represents the 95% confidence interval of the prediction. The strength of the association between anticipated guilt and moral judgments was significantly weaker for those adolescents with higher levels of CU traits.

To examine whether CU traits moderate the relation between anticipated guilt and wrongness judgments, a separate linear mixed model, adding an interaction term between anticipated guilt and CU traits, was fitted (see Table 4). All predictor variables were grand mean centered. ICU score moderated the relation between anticipated guilt and wrongness ratings (β = −0.01, *p* < 0.001) revealing a weaker association between those components of moral judgment in participants with higher CU levels. Adolescents with lower levels of CU traits showed a steep increase in the wrongfulness ratings as the ratings of anticipated guilt augmented, whilst this was not observed in those with high levels of CU traits (Figure 3).

**Table 4:** **Linear mixed model for the relation between anticipated guilt and wrongness judgments moderated by CU traits**.

	**Wrongness ratings**		
**Fixed effects**	**β**	**SE**	***p-value***
Intercept	16.36	0.14	< 0.001
ICU score	−0.03	0.02	0.02
Guilt	0.6	0.02	< 0.001
Guilt*ICU score	−0.01	0.00	< 0.001
**Random effects**	***SD*** **(CI)**		
Scenario (intercept)	0.6 (0.34–0.87)		
Participant (intercept)	2.7 (2.27–3.10)		
Residual	1.0 (0.35–2.85)		
Marginal R^2^	0.427		
AIC	5964.85		

β, unstandardized regression coefficient; SE, standard error; ICU, Inventory of Callous-Unemotional traits; CI, confidence interval; AIC, Akaike Information Criterion.

### Is the Strength of the Association Between Anticipated Guilt and Response Times to Wrongness Judgments Moderated by CU Traits?

Finally, and to inspect whether higher anticipated guilt has a facilitating effect on wrongness judgments and whether this is impacted by CU traits, we examined if CU traits moderate the relation between anticipated guilt ratings and wrongness judgments' RTs. A separate linear mixed model was fitted, adding an interaction term between anticipated guilt and CU traits (see Table 5). Higher levels of anticipated guilt were associated with lower response times (β = −35.05, *p* < 0.001) to wrongness judgments. CU traits did not moderate the association between anticipated guilt and response time to wrongness judgements (*p* = 0.71).

**Table 5:** **Linear mixed model for relations between anticipated guilt and wrongness judgments' RTs moderated by CU traits.**.

	**Wrongness RTs**		
**Fixed effects**	**β**	**SE**	***p-value***
Intercept	1307.64	32.75	<0.001
ICU score	11.34	3.41	< 0.001
Guilt	−35.05	5.26	< 0.001
Guilt*ICU score	−0.30	0.82	0.709
**Random effects**	***SD*** **(CI)**		
Scenario (intercept)	127.9 (81.43–200.98)		
Participant (intercept)	653.8 (20.90–20450.47)		
Residual	246.5 (0.01–80769.77)		
Marginal R^2^	0.061		
AIC	19145.91		

β, unstandardized regression coefficient; SE, standard error; ICU, Inventory of Callous-Unemotional traits; CI, confidence interval; AIC, Akaike Information Criterion.

## Discussion

The presence of high levels of CU traits, a constellation of personality traits marked by blunted affect, uncaring behavior and disregard for others [e.g., (3)], in youth is associated with increased antisocial behavior, even in the absence of a diagnosis of conduct disorder (5, 12). CU traits are considered to be developmental precursors of core affective-interpersonal aspects of psychopathy, a disorder marked by serious and persistent antisocial behavior (2, 4, 28, 64, 65). A growing body of research in adult samples suggests that moral dysfunction in psychopathy may stem from a disruption of the affective components of moral processing rather than from an inability to compute moral judgments *per se*. Here, we present data on a novel task of everyday moral processing and test if the dissociation between affective and cognitive dimensions of moral processing linked to psychopathic traits in adulthood is also observed in relation to CU traits in adolescence, in a typically-developing adolescent sample. In this task, adolescents were presented with personal everyday moral transgression scenarios and were asked to rate how much guilt they would feel and how wrong it would be if they performed such actions. We found that CU traits were negatively associated with both anticipated guilt and wrongness appraisals to the transgressions; adolescents with higher CU traits anticipated feeling less guilt and made less wrongfulness judgments. However, we also found that higher levels of CU traits were associated with increased dissociation between anticipated guilt and moral judgment. That is, in contrast to those with low levels of CU traits, adolescents with high levels of CU traits did not show a steeper increase in wrongness ratings as the ratings of anticipated guilt augmented.

Our key finding relates to the moderating role of CU traits in the association between anticipated guilt and moral judgment. Contemporary perspectives of human morality emphasize the role of moral emotions on moral judgement, in particular in care-based moral judgments (15, 18, 19). Moral emotions are thought to play a fundamental role on the development of judgments about moral situations (66, 67). In our sample, CU traits were negatively associated with the strength of the association between guilt and wrongness judgments. For adolescents with higher levels of these traits, moral emotions did not seem to play such an important role on moral appraisals. This is interesting, because it suggests that the dissociation between moral emotions and moral judgment linked to psychopathic traits may already be present during adolescence and evolve through adulthood. Findings from neuroimaging research on moral processing in adolescents with high CU traits, albeit sparse, are in-line with this idea. CU traits have been found to be associated with diminished amygdala response and connectivity with prefrontal regions during moral judgment (47, 48). The amygdala is a brain region critical for affective processing and this pattern of diminished response might reflect reduced affective input during moral judgement. It is possible that the moderating role of CU traits in the association between anticipated guilt and wrongness appraisals reflects a lack of “affective coloring” in moral judgment in adolescents with high levels of CU.

In line with findings from adult research [e.g., (30, 31, 35)] and a recent study with a youth community sample (45), we further found that adolescents with higher CU traits reported anticipating less guilt when imagining themselves performing everyday actions that cause harm to other people, and took longer to make these evaluations. This finding agrees with recent literature reporting emotional hypo-responsivity of children with high levels of CU traits, in particular to others' stress, manifested at behavioral, cognitive and neural levels (see 3, 49 for recent reviews). Our findings also revealed that adolescents with higher levels of CU traits judge everyday moral transgressions as less wrongful. This is in contrast to the majority of findings from adult studies indicating that individuals with high levels of psychopathic traits have apparently intact moral judgment abilities. For example, the endorsement of actions in moral dilemmas seems to be similar for adult psychopaths and controls (32, 68) and does not seem to vary with levels of psychopathic traits in community samples (30, 31). Additionally, psychopathic traits do not seem to be associated with moral wrongness judgments of aggressive behavior (69), nor of everyday moral situations (35). Findings from child and adolescent samples are sparser and less consistent. For example, Marsh et al. (48) did not find behavioral differences between youth with high levels of psychopathic traits and matched controls when making explicit moral judgments. Harenski et al. (47) found that psychopathic traits in a sample of incarcerated youth were negatively associated with ratings of violation severity of pictures with moral content. But, psychopathic traits were also negatively associated with ratings of non-moral but still unpleasant pictures, which might indicate a lack of specificity to moral content.

It has been proposed that, in the absence of an emotional response to moral transgressions, individuals with high levels of psychopathic traits use alternative cognitive strategies to process moral judgments (61). Individuals with high levels of psychopathy seem to know (and apply) the rules that are relevant to make moral judgements but do seem to do so without using “standard” affective routes that are taken by those with low levels of these traits (32, 68, 70). This point is supported by our last analysis. We found a negative association between guilt anticipation and response time to wrongness judgements. Participants took less time to provide their wrongness ratings when anticipated guilt was high. Hence, higher response time when making a moral appraisal in participants higher in CU may reflect the lack of emotional loading when judging the scenario and the reliance on other cues to provide a response. Our results thus suggest that differences in appraisals of wrongness are still present in adolescents with high CU, alongside difficulties in making these appraisals, but a weaker role of emotion on moral judgement is also already observed.

Additionally, it is possible that adults with high levels of psychopathic traits display typical moral judgment due to an intact ability to understand the thoughts of others (36, 44), as well as others' expectations (71). This would enable adults with high levels of psychopathic traits to respond to moral tasks and questionnaires in the same way they think other people would, thus engaging in successful impression management. Social cognition skills are not yet fully developed during adolescence [see (72) for a review]. The ability to understand the thoughts of others and flexibility in taking another's perspective into account seems to be still developing in late adolescence and early adulthood (73). Plus, recent evidence suggests that, although adolescents with higher levels of CU traits do not seem to be less able than their peers to infer the thoughts of others, they seem to be less prone to take others' thoughts into account, at least in a spontaneous and effortless manner (74). This could explain why more lenient moral appraisals are observed in adolescents with higher levels of CU but not in adults with higher levels of psychopathic traits. This could also explain why adolescents with higher levels of CU take longer to make such judgments.

The present work provides relevant evidence regarding early signs of a dissociation between affective (i.e., anticipated guilt) and cognitive (i.e., wrongness judgments) dimensions of moral behavior in adolescents with high levels of CU traits. However, it is important to bear in mind that the lack of a comparison group of adults precluded us from examining if and how the moderation of the association between guilt and moral judgments by psychopathy changes/increases with age. Future cross-sectional and, critically, longitudinal research is required to confirm an increased dissociation between different components of moral processing from adolescence to adulthood for those with high levels of psychopathic traits. A larger and mixed-gender sample would also allow us more power to detect possible smaller effects and also to test if the links found between moral processing components and CU traits are similar for boys and girls or whether there are gender-specific differences, in line with recent research examining gender differences in moral emotions (75). It should also be noted that cognitive ability was not controlled for in this study. Research findings on how intelligence interacts with psychopathy and antisocial behavior is diverse, with studies suggesting that IQ is likely a protective factor against antisocial behavior (76) whilst others indicate that enhanced verbal abilities potentiate the relation between callous-unemotional traits and violent juvenile offending (77). We have not included a measure of cognitive ability in the current study for a number of reasons: there is limited evidence of the role of IQ on processing of everyday moral transgressions in adolescents (78); our sample was comprised of typically developing adolescents from a regular high school and we did not expect to find adolescents outside the typical range of IQ; and we had limited testing time imposed by the school. Future work should investigate whether IQ does play a role on the processing of both cognitive and emotional aspects of everyday transgressions in a large adolescent sample with varying levels of IQ, and also address whether and how cognitive abilities modify the effect we found in the present work.

Despite these limitations, this study presents important new evidence of how, in typically developing adolescents, the presence of CU traits moderates the association between affective and cognitive components of moral processing. Our findings are in line with the notion that evidence gathered from community studies often reflect the findings of forensic and clinical investigations (79) and support the dimensional approach to psychopathy suggesting that correlates of psychopathic traits in adults and CU traits in children and youth are present in a continuum in the population [see (80), for a review]. Plus, they add evidence to the view that CU traits are strongly linked to impairments in emotional functioning, and that these impairments may play an important role in explaining moral dysfunction in youth with high psychopathic traits (81). The present findings make important contributions to further characterize and comprehend the cognitive-affective processing deficits that may underlie atypical moral processing in youth who are at heightened risk for antisocial behavior and psychopathy in adulthood.

## Data Availability Statement

The raw data supporting the conclusions of this article will be made available by the authors, without undue reservation.

## Ethics Statement

The studies involving human participants were reviewed and approved by Ethics Committee of University of Minho, Braga, Portugal (SECVS131/2016). Written informed consent to participate in this study was provided by the participants' legal guardian/next of kin.

## Author Contributions

AS-C, EV, CS, and AS: conceptualization. SF, MV, and AS-C: statistical analyses. AS-C, MV, EV, and CS: writing—original draft. AS-C: funding acquisition. All authors: writing—review & editing, contributed to the article, and approved the submitted version.

## Conflict of Interest

The authors declare that the research was conducted in the absence of any commercial or financial relationships that could be construed as a potential conflict of interest.
