# Supplementary material for: Callous-Unemotional Traits Moderate Anticipated Guilt and Wrongness Judgments to Everyday Moral Transgressions in Adolescents
Source: Front Psychiatry. 2021 Mar 5;12:625328. doi: 10.3389/fpsyt.2021.625328 (PMC7982950; doi:10.3389/fpsyt.2021.625328)
Supplement: Supplementary file 1 [file Data_Sheet_1.PDF]

Supplemental Material

Supplemental Table 1.

Paired-Samples T Tests comparing HTO vs. HTS scenarios for Wrongness and Guilt ratings.

	Wrongness Judgements						Anticipated Guilt					
	**HTO**	**HTS**	***t***	***df***	***p***	**Cohen's *d***	**HTO**	**HTS**	***t***	***df***	***p***	**Cohen's *d***
**Scenario 1**	17.21	12.04	5.87	46	***	1.01	17.55	11.77	6.90	46	***	0.86
**Scenario 2**	17.30	10.57	7.15	46	***	0.94	17.51	12.17	6.44	46	***	1.04
**Scenario 3**	17.85	9.21	9.51	46	***	2.26	18.62	5.83	15.49	46	***	1.39
**Scenario 4**	17.06	4.57	15.97	46	***	2.42	17.32	4.57	16.58	46	***	2.33
**Scenario 5**	15.94	9.09	7.86	44	***	1.37	17.23	9.19	9.41	46	***	1.17
**Scenario 6**	17.80	5.65	13.03	45	***	2.30	18.35	4.67	15.57	45	***	1.49
**Scenario 7**	13.59	11.09	2.76	44	**	0.41	14.24	12.34	2.75	43	**	0.41
**Scenario 8**	15.91	8.85	6.40	45	***	1.25	16.78	8.93	8.51	45	***	0.96
**Scenario 9**	15.59	12.35	3.61	45	***	1.57	15.87	7.64	10.15	41	***	0.53
**Scenario 10**	15.18	9.25	5.72	43	***	2.49	15.60	2.77	16.49	43	***	0.88
**Scenario 11**	15.73	12.05	3.26	43	**	1.24	17.18	9.27	8.31	43	***	0.48

**Scenario 12**	12.77	8.64	4.73	46	***	1.20	13.79	6.83	8.12	45	***	0.69
**Scenario 13**	15.48	9.58	5.32	44	***	2.01	16.70	5.70	13.64	45	***	0.81
**Scenario 14**	15.43	9.46	5.77	45	***	1.16	15.80	8.76	8.02	44	***	0.85
**Scenario 15**	14.87	5.75	10.07	43	***	1.69	15.35	4.64	11.31	44	***	1.52
**Scenario 16**	12.17	10.63	1.63	45	ns	0.48	13.04	10.07	3.26	45	**	0.25
**Scenario 17**	13.59	8.62	4.86	44	***	1.23	15.17	7.02	8.17	43	***	0.73
**Scenario 18**	17.28	10.47	7.77	44	***	1.00	18.17	11.47	6.73	44	***	1.16
**Scenario 19**	15.96	7.93	8.14	44	***	1.43	16.26	7.89	9.67	45	***	1.21
**Scenario 20**	15.64	10.50	5.95	43	***	1.23	16.56	10.07	7.98	40	***	0.90
**Scenario 21**	17.72	6.94	12.24	46	***	2.44	18.49	4.94	16.70	46	***	1.79
**Scenario 22**	18.02	9.09	8.24	46	***	1.41	18.40	9.30	9.54	45	***	1.20
**Scenario 23**	16.98	7.22	10.12	45	***	2.72	17.38	3.13	18.43	45	***	1.49
**Scenario 24**	15.78	5.09	10.77	44	***	1.46	16.33	5.78	9.65	44	***	1.63
**Scenario 25**	15.26	6.63	9.44	45	***	1.49	14.83	5.52	10.09	45	***	1.39
**Scenario 26**	13.96	6.56	7.37	44	***	1.27	14.00	5.84	8.75	44	***	1.10
**Scenario 27**	13.09	9.42	3.89	44	***	1.09	13.87	7.91	7.28	44	***	0.60

Notes. HTO = harm-to-other; HTS = harm-to-self; ns = nonsignificant; * $p < .05$; ** $p < .01$; *** $p < .001$

Supplemental Table 2.

Linear mixed models of Anticipated Guilt and Moral Wrongness ratings including the effects of CU traits, excluding potential outlier observations in anticipated guilt (RT = 39 ms).

	Guilt ratings			Wrongness ratings		
**Fixed effects**	**β**	**SE**	***p-value***	**β**	**SE**	***p-value***
Intercept	15.81	0.30	< .001	16.44	0.29	< .001
ICU score	-0.18	0.02	< .001	-0.15	0.02	< .001
**Random effects**	***SD* (CI)**			***SD* (CI)**		
Scenario (intercept)	1.39 (1.01 - 1.90)			1.38 (1.01 - 1.88)		
Participant (intercept)	3.57 (2.65 - 4.80)			3.33 (2.62 - 4.22)		
Residual	1.37 (0.19 - 9.95)			1.28 (0.17 - 6.19)		
Marginal R^2	0.08			0.06		
AIC	6675.25			6512.70		

Note. β = unstandardized regression coefficient; SE = standard error; ICU = Inventory of Callous-Unemotional traits; CI = confidence interval; AIC = Akaike Information Criterion

Supplemental Table 3.

Linear mixed models of Anticipated Guilt and Moral Wrongness ratings including the effects of CU traits, excluding potential outlier observations in anticipated guilt (RT = 39 ms & RT = 3891 ms).

	Guilt ratings			Wrongness ratings		
**Fixed effects**	**β**	**SE**	***p-value***	**β**	**SE**	***p-value***
Intercept	15.81	0.30	< .001	16.44	0.29	< .001
ICU score	-0.18	0.02	< .001	-0.15	0.02	< .001
**Random effects**	***SD* (CI)**			***SD* (CI)**		
Scenario (intercept)	1.42 (1.03 - 1.96)			1.41 (1.03 - 1.93)		
Participant (intercept)	3.57 (2.74 - 4.66)			3.33 (2.71 - 4.09)		
Residual	1.37 (0.23 - 8.13)			1.28 (0.33 - 4.91)		
Marginal R^2	0.08			0.06		
AIC	6677.51			6515.30		

Note. β = unstandardized regression coefficient; SE = standard error; ICU = Inventory of Callous-Unemotional traits; CI = confidence interval; AIC = Akaike Information Criterion

Supplemental Table 4.

Linear mixed models of Anticipated Guilt and Wrongness RTs including the effects of CU traits, excluding potential outlier observations in anticipated guilt (RT = 39 ms).

	Guilt RTs			Wrongness RTs		
**Fixed effects**	**β**	**SE**	***p-value***	**β**	**SE**	***p-value***
Intercept	1443.03	36.71	< .001	1310.55	36.67	< .001
ICU Total score	15.22	3.27	< .001	17.38	3.26	< .001
**Random effects**	***SD* (CI)**			***SD* (CI)**		
Scenario (intercept)	155.00 (104.18 - 230.45)			154.76 (104.03 - 230.21)		
Participant (intercept)	664.49 (10.52 - 41971.24)			664.47 (55.07 - 8016.89)		
Residual	251.40 (0.01 - 923072.00)			251.47 (0.01 - 886635.00)		
Marginal R^2	0.02			0.03		
AIC	19172.04			19171.89		

Note. β = unstandardized regression coefficient; SE = standard error; ICU = Inventory of Callous-Unemotional traits; CI = confidence interval; AIC = Akaike Information Criterion

Supplemental Table 5.

Linear mixed models of Anticipated Guilt and Wrongness RTs including the effects of CU traits, excluding potential outlier observations in anticipated guilt (RT = 39 ms & RT = 3891 ms).

	Guilt RTs			Wrongness RTs		
**Fixed effects**	**β**	**SE**	***p-value***	**β**	**SE**	***p-value***
Intercept	1441.02	36.10	< .001	1310.54	36.68	< .001
ICU Total score	15.42	3.26	< .001	17.39	3.27	< .001
**Random effects**	***SD* (CI)**			***SD* (CI)**		
Scenario (intercept)	151.47 (101.32 - 226.39)			154.75 (104.01 - 230.22)		
Participant (intercept)	662.01 (32.32 - 13561.71)			664.75 (20.22 - 20853.65)		
Residual	251.40 (0.01 - 361757.30)			251.63 (0.01 - 970218.00)		
Marginal R^2	0.02			0.03		
AIC	19146.45			19156.89		

Note. β = unstandardized regression coefficient; SE = standard error; ICU = Inventory of Callous-Unemotional traits; CI = confidence interval; AIC = Akaike Information Criterion

Supplemental Table 6.

Linear mixed model for the relation between anticipated guilt and wrongness judgments moderated by CU traits, excluding potential outlier observations in anticipated guilt (RT = 39 ms).

	**Wrongness ratings**		
**Fixed effects**	**β**	**SE**	***p-value***
Intercept	16.37	0.14	< .001
ICU score	-0.03	0.01	.04
Guilt	0.60	0.02	< .001
Guilt*ICU score	-0.01	0.00	< .001
**Random effects**	***SD* (CI)**		
Scenario (intercept)	0.53 (0.33 - 0.84)		
Participant (intercept)	2.65 (2.39 - 2.95)		
Residual	1.00 (0.50 - 1.96)		
Marginal R^2	0.45		
AIC	5936.41		

Note. β = unstandardized regression coefficient; SE = standard error; ICU = Inventory of Callous-Unemotional traits; CI = confidence interval; AIC = Akaike Information Criterion

Supplemental Table 7.

Linear mixed model for the relation between anticipated guilt and wrongness judgments moderated by CU traits, excluding potential outlier observations in anticipated guilt (RT = 39 ms & RT = 3891 ms).

	Wrongness ratings		
**Fixed effects**	**β**	**SE**	***p-value***
Intercept	16.36	0.13	< .001
ICU score	-0.03	0.01	.04
Guilt	0.60	0.02	< .001
Guilt*ICU score	-0.01	0.00	< .001
**Random effects**	***SD* (CI)**		
Scenario (intercept)	0.53 (0.33 - 0.84)		
Participant (intercept)	2.65 (2.39 - 2.82)		
Residual	1.00 (0.78 - 1.28)		
Marginal R^2	0.45		
AIC	5032.17		

Note. β = unstandardized regression coefficient; SE = standard error; ICU = Inventory of Callous-Unemotional traits; CI = confidence interval; AIC = Akaike Information Criterion

Supplemental Table 8.

Linear mixed model for relations between anticipated guilt and wrongness judgments' RTs moderated by CU traits, excluding potential outlier observations in anticipated guilt (RT = 39 ms).

	Wrongness RTs		
**Fixed effects**	**β**	**SE**	***p-value***
Intercept	1308.40	32.70	< .001
ICU score	11.31	3.41	.002
Guilt	-35.10	5.26	< .001
Guilt*ICU score	-0.31	0.82	.70
**Random effects**	***SD* (CI)**		
Scenario (intercept)	127.48 (811.11 - 200.61)		
Participant (intercept)	654.00 (26.36 - 16226.09)		
Residual	246.54 (0.01 - 158904.10)		
Marginal R^2	0.06		
AIC	19130.74		

Note. β = unstandardized regression coefficient; SE = standard error; ICU = Inventory of Callous-Unemotional traits; CI = confidence interval; AIC = Akaike Information Criterion.

Supplemental Table 9.

Linear mixed model for relations between anticipated guilt and wrongness judgments' RTs moderated by CU traits, excluding potential outlier observations in anticipated guilt (RT = 39 ms & RT = 3891 ms).

	Wrongness RTs		
**Fixed effects**	**β**	**SE**	***p-value***
Intercept	1308.40	32.71	< .001
ICU score	11.31	3.41	.002
Guilt	-35.02	5.26	< .001
Guilt*ICU score	-0.31	0.81	.70
**Random effects**	***SD* (CI)**		
Scenario (intercept)	127.48 (80.99 - 200.66)		
Participant (intercept)	654.28 (1.92 - 222110.80)		
Residual	246.70 (0.01 - 156390.20)		
Marginal R^2	0.06		
AIC	19115.79		

Note. β = unstandardized regression coefficient; SE = standard error; ICU = Inventory of Callous-Unemotional traits; CI = confidence interval; AIC = Akaike Information Criterion.

Supplemental Table 10.

Linear mixed models of Anticipated Guilt and Moral Wrongness ratings including the effects of CU traits and age.

	Guilt ratings			Wrongness ratings		
**Fixed effects**	**β**	**SE**	***p-value***	**β**	**SE**	***p-value***
Intercept	15.81	0.30	< .001	16.44	0.29	< .001
ICU score	-0.18	0.02	< .001	-0.15	0.02	< .001
Age	0.09	0.13	.46	0.27	0.12	.02
**Random effects**	***SD* (CI)**			***SD* (CI)**		
Scenario (intercept)	1.39 (1.01 – 1.91)			1.38 (1.01 - 1.88)		
Participant (intercept)	3.57 (2.79 – 4.57)			3.32 (1.58 – 6.97)		
Residual	1.37 (0.127- 7.07)			1.28 (0.01 – 18.56)		
Marginal R^2	0.08			0.06		
AIC	6681.37			6513.82		

Note. β = unstandardized regression coefficient; SE = standard error; ICU = Inventory of Callous-Unemotional traits; CI = confidence interval; AIC = Akaike Information Criterion

Supplemental Table 11.

Linear mixed models of Anticipated Guilt and Moral Wrongness ratings including the effects of CU traits and age, excluding potential outlier observations in anticipated guilt (RT = 39 ms).

	Guilt ratings			Wrongness ratings		
**Fixed effects**	**β**	**SE**	***p-value***	**β**	**SE**	***p-value***
Intercept	15.81	0.30	< .001	16.44	0.29	< .001
ICU score	-0.18	0.02	< .001	-0.15	0.02	< .001
Age	0.09	0.13	.46	0.27	0.12	.02
**Random effects**	***SD* (CI)**			***SD* (CI)**		
Scenario (intercept)	1.38 (1.01 - 1.90)			1.38 (1.01 - 1.88)		
Participant (intercept)	3.57 (2.93 - 4.34)			3.32 (2.41 - 4.60)		
Residual	1.37 (0.38 - 4.98)			1.28 (0.15 - 10.97)		
Marginal R^2	0.08			0.06		
AIC	6676.69			6509.36		

Note. β = unstandardized regression coefficient; SE = standard error; ICU = Inventory of Callous-Unemotional traits; CI = confidence interval; AIC = Akaike Information Criterion

Supplemental Table 12.

Linear mixed models of Anticipated Guilt and Moral Wrongness ratings including the effects of CU traits and age, excluding potential outlier observations in anticipated guilt (RT = 39 ms & RT = 3891 ms).

	Guilt ratings			Wrongness ratings		
**Fixed effects**	**β**	**SE**	***p-value***	**β**	**SE**	***p-value***
Intercept	15.81	0.30	< .001	16.44	0.29	< .001
ICU score	-0.17	0.02	< .001	-0.14	0.02	< .001
Age	0.10	0.13	.44	0.27	0.12	.02
**Random effects**	***SD* (CI)**			***SD* (CI)**		
Scenario (intercept)	1.38 (1.01 - 1.91)			1.38 (1.01 - 1.88)		
Participant (intercept)	3.57 (2.88 - 4.42)			3.32 (2.54 - 4.34)		
Residual	1.37 (0.33 - 5.92)			1.28 (0.22 - 7.51)		
Marginal R^2	0.08			0.06		
AIC	6671.71			6504.94		

Note. β = unstandardized regression coefficient; SE = standard error; ICU = Inventory of Callous-Unemotional traits; CI = confidence interval; AIC = Akaike Information Criterion

Supplemental Table 13.

Linear mixed models of Anticipated Guilt and Wrongness RTs including the effects of CU traits and age.

	Guilt RTs			Wrongness RTs		
**Fixed effects**	β	SE	*p-value*	β	SE	*p-value*
Intercept	1441.94	36.88	< .001	1310.16	36.82	< .001
ICU Total score	14.98	3.28	< .001	16.49	3.28	< .001
Age	-26.04	23.22	.26	-62.01	23.13	.01
**Random effects**	***SD* (CI)**			***SD* (CI)**		
Scenario (intercept)	155.90 (105.1 – 231.39)			155.90 (105.07 – 231.17)		
Participant (intercept)	664.60 (0.33 – 13471.20)			662.15 (7.16 – 61231.21)		
Residual	251.60 (0.01 – 296416.20)			250.70 (0.01 - 129776)		
Marginal R^2	0.02			0.03		
AIC	19190.72			19181.96		

Note. β = unstandardized regression coefficient; SE = standard error; ICU = Inventory of Callous-Unemotional traits; CI = confidence interval; AIC = Akaike Information Criterion

Supplemental Table 14.

Linear mixed models of Anticipated Guilt and Wrongness RTs including the effects of CU traits and age, excluding potential outlier observations in anticipated guilt (RT = 39 ms).

	Guilt RTs			Wrongness RTs		
**Fixed effects**	**β**	**SE**	***p-value***	**β**	**SE**	***p-value***
Intercept	1443.03	36.71	< .001	1310.55	36.67	< .001
ICU Total score	14.81	3.29	< .001	16.44	5.01	< .001
Age	-26.42	23.21	.26	-62.13	23.15	.01
**Random effects**	***SD* (CI)**			***SD* (CI)**		
Scenario (intercept)	155.13 (104.36 - 230.61)			155.26 (104.5 - 230.59)		
Participant (intercept)	664.12 (24.44 - 18044.62)			662.40 (15.68 - 27977.18)		
Residual	251.40 (0.01 - 25537.20)			250.80 (0.01 - 510567)		
Marginal R^2	0.02			0.03		
AIC	19172.74			19166.69		

Note. β = unstandardized regression coefficient; SE = standard error; ICU = Inventory of Callous-Unemotional traits; CI = confidence interval; AIC = Akaike Information Criterion

Supplemental Table 15.

Linear mixed models of Anticipated Guilt and Wrongness RTs including the effects of CU traits and age, excluding potential outlier observations in anticipated guilt (RT = 39 ms & RT = 3891 ms).

	**Guilt RTs**			**Wrongness RTs**		
**Fixed effects**	**β**	**SE**	***p-value***	**β**	**SE**	***p-value***
Intercept	1441.09	36.12	< .001	1310.53	36.74	< .001
ICU Total score	14.94	3.27	< .001	16.45	3.28	< .001
Age	-30.82	23.15	.26	-62.49	23.19	.01
**Random effects**	***SD*** **(CI)**			***SD*** **(CI)**		
Scenario (intercept)	151.59 (101.45 - 226.49)			155.23 (104.5 - 230.58)		
Participant (intercept)	661.55 (27.11 - 16139.47)			662.66 (58.64 - 7488.27)		
Residual	250.32 (0.01 - 12235.70)			250.87 (0.01 - 557319.70)		
Marginal R^2	0.02			0.03		
AIC	19146.67			19151.64		

Note. β = unstandardized regression coefficient; SE = standard error; ICU = Inventory of Callous-Unemotional traits; CI = confidence interval; AIC = Akaike Information Criterion

Supplemental Table 16.

Linear mixed model for the relation between anticipated guilt and wrongness judgments moderated by CU traits and age

	Wrongness ratings		
**Fixed effects**	**β**	**SE**	***p-value***
Intercept	16.37	0.14	< .001
ICU score	-0.03	0.01	.04
Guilt	0.60	0.02	< .001
Guilt*ICU score	-0.01	0.00	< .001
Age	0.18	0.09	.06
**Random effects**	***SD* (CI)**		
Scenario (intercept)	0.55 (0.35 - 0.87)		
Participant (intercept)	2.65 (2.5 -2.83)		
Residual	1.00 (0.71 – 1.41)		
Marginal R^2	0.45		
AIC	5966.17		

Note. β = unstandardized regression coefficient; SE = standard error; ICU = Inventory of Callous-Unemotional traits; CI = confidence interval; AIC = Akaike Information Criterion

Supplemental Table 17.

Linear mixed model for the relation between anticipated guilt and wrongness judgments moderated by CU traits and age, excluding potential outlier observations in anticipated guilt (RT = 39 ms).

	Wrongness ratings		
**Fixed effects**	**β**	**SE**	***p-value***
Intercept	16.37	0.14	< .001
ICU score	-0.03	0.01	.04
Guilt	0.60	0.02	< .001
Guilt*ICU score	-0.01	0.00	< .001
Age	0.18	0.09	.06
**Random effects**	***SD* (CI)**		
Scenario (intercept)	0.53 (0.34 - 0.84)		
Participant (intercept)	2.65 (2.47 - 2.84)		
Residual	1.00 (0.69 - 1.45)		
Marginal R^2	0.45		
AIC	5934.83		

Note. β = unstandardized regression coefficient; SE = standard error; ICU = Inventory of Callous-Unemotional traits; CI = confidence interval; AIC = Akaike Information Criterion

Supplemental Table 18.

Linear mixed model for the relation between anticipated guilt and wrongness judgments moderated by CU traits and age, excluding potential outlier observations in anticipated guilt (RT = 39 ms & RT = 3891 ms).

	Wrongness ratings		
**Fixed effects**	**β**	**SE**	***p-value***
Intercept	16.36	0.13	< .001
ICU score	-0.03	0.01	.04
Guilt	0.60	0.02	< .001
Guilt*ICU score	-0.01	0.00	< .001
Age	0.18	0.09	.06
**Random effects**	***SD* (CI)**		
Scenario (intercept)	0.53 (0.34 - 0.84)		
Participant (intercept)	2.65 (2.47 - 2.83)		
Residual	1.00 (0.69 - 1.45)		
Marginal R^2	0.45		
AIC	5930.71		

Note. β = unstandardized regression coefficient; SE = standard error; ICU = Inventory of Callous-Unemotional traits; CI = confidence interval; AIC = Akaike Information Criterion

Supplemental Table 19.

Linear mixed model for relations between anticipated guilt and wrongness judgments' RTs moderated by CU traits and age.

	**Wrongness RTs**		
**Fixed effects**	**β**	**SE**	***p-value***
Intercept	1305.77	32.90	< .001
ICU score	10.69	3.41	.002
Guilt	-34.45	5.25	< .001
Guilt*ICU score	-0.57	0.82	.49
Age	-60.65	22.96	.01
**Random effects**	***SD* (CI)**		
Scenario (intercept)	128.99 (82.44 - 201.86)		
Participant (intercept)	651.73 (16.62 – 25565.11)		
Residual	245.82 (0.01 – 390832.10)		
Marginal R^2	0.07		
AIC	19140.92		

Note. β = unstandardized regression coefficient; SE = standard error; ICU = Inventory of Callous-Unemotional traits; CI = confidence interval; AIC = Akaike Information Criterion

Supplemental Table 20.

Linear mixed model for relations between anticipated guilt and wrongness judgments' RTs moderated by CU traits and age, excluding potential outlier observations in anticipated guilt (RT = 39 ms).

	Wrongness RTs		
**Fixed effects**	**β**	**SE**	***p-value***
Intercept	1306.17	32.83	< .001
ICU score	10.65	3.41	.002
Guilt	-34.42	5.25	< .001
Guilt*ICU score	-0.58	0.82	.47
Age	-60.65	22.97	.01
**Random effects**	***SD* (CI)**		
Scenario (intercept)	128.52 (81.99 - 201.46)		
Participant (intercept)	651.98 (17.62 - 24124.39)		
Residual	245.89 (0.01 - 260583.40)		
Marginal R^2	0.07		
AIC	19125.73		

Note. β = unstandardized regression coefficient; SE = standard error; ICU = Inventory of Callous-Unemotional traits; CI = confidence interval; AIC = Akaike Information Criterion.

Supplemental Table 21.

Linear mixed model for relations between anticipated guilt and wrongness judgments' RTs moderated by CU traits and age, excluding potential outlier observations in anticipated guilt (RT = 39 ms & RT = 3891 ms).

	Wrongness RTs		
**Fixed effects**	**β**	**SE**	***p-value***
Intercept	1306.11	32.85	< .001
ICU score	10.66	3.41	.002
Guilt	-34.39	5.26	< .001
Guilt*ICU score	-0.59	0.82	.47
Age	-60.03	23.02	.01
**Random effects**	***SD* (CI)**		
Scenario (intercept)	128.53 (81.98 - 201.49)		
Participant (intercept)	652.25 (5.54 - 76864.55)		
Residual	245.99 (0.01 - 89927.4)		
Marginal R^2	0.07		
AIC	19110.76		

Note. β = unstandardized regression coefficient; SE = standard error; ICU = Inventory of Callous-Unemotional traits; CI = confidence interval; AIC = Akaike Information Criterion.
